# Supplementary material for: bp: Blood pressure analysis in R
Source: PLoS One. 2022 Sep 9;17(9):e0268934. doi: 10.1371/journal.pone.0268934 (PMC9462781; doi:10.1371/journal.pone.0268934)
Supplement: Attachment — Submitted filename: ResponseToReviews.pdf [file pone.0268934.s001.pdf]

Response to Reviews for "bp: Blood Pressure Analysis in R"

Thank you for the opportunity to revise our manuscript. We are grateful to the Editor and the reviewers for the comments and suggestions that helped significantly improve both the manuscript and the software. Our revisions are detailed below. We use *cursive font* for your comments, and blue font for our response.

R.1 Reviewer 1 Comments

In this paper, the authors present a program for analyzing continuous BP, home BP, and ambulatory BP data. The program is developed in R language and provides data analysis and sample datasets functions. In general, although not novel, the idea is interesting since the software is provided as an open-source program.

Thank you for your summary and feedback.

1. *However, as correctly stated by the authors, the user needs to know how the R language works.*

   The reviewer is correct that familiarity with the R programming language is currently a pre-requisite for the use of the package, a limitation that we acknowledge in the discussion. However, this limitation is inherent to any programming-based software solution, e.g. SAS, Python, etc. While software with a graphical user interface (GUI), like SPSS or available proprietary software for BP data (e.g. AccuWin Pro), is undoubtedly easier to use for researchers with limited programming experience, the main disadvantage of such software is lack of automation and reproducibility. Such platforms (e.g., SPSS, Excel, GUI-only proprietary solutions) are less desirable for users who want to create reproducible scripts for all data processing and metric calculation steps. Our choice of the R language over other scripting language alternatives (e.g. Python, SAS) is motivated by its accessibility as an open-source platform, and its popularity (Masuadi et al., 2021; Muenchen, 2012). We have revised the Introduction to better justify our choice of R.

   Ideally, we would like to have a solution that combines the computational advantages of the R language with the user-friendliness of graphical user interfaces, precisely to eliminate the need for the user to know R. Web applications called Shiny Apps (Chang et al., 2020) address this issue by providing users with a graphical user interface that relies on underlying R scripts. These Shiny Apps are dynamic and interactive, giving the user the flexibility to slice the data into various partitions with the click of a mouse, with the outputs automatically reflecting such changes. It is our goal to develop such a Shiny App for the `bp` package to further increase package accessibility. At the moment, we have a prototype version of the app with significantly reduced functionality compared to the full `bp` package; we plan to continue working on the app to make a full release in the future. The work on the app, however, is still incomplete and is outside the scope of current manuscript. We have revised the Discussion

to further highlight possibilities of integrating a Shiny App with the package and to outline it as future work.

2. *Usually, BP data sets are analyzed with statistical packages such as SAS and SPSS. These programs are not free, at variance with the proposed program. However, they are beneficial in analyzing BP data by constructing simple lines of code. The authors should discuss the advantage of their program compared to these packages. The authors should have validated results obtained with their code with any known validated and certified software.*

   Thank you for highlighting other software that was not mentioned in the paper. We agree that SAS and SPSS commercial statistical software packages are often used in analyses of blood pressure data. We chose to use R software platform over existing alternatives due to several reasons. First, R is a script-based programming language, which allows the creation of reproducible scripts for all BP data processing and metric calculation steps. This is not possible with point and click software solutions, such as Excel or SPSS statistical software. Second, R is gaining popularity in analyes of blood pressure data, being used for example in Ahmed et al. (2015); Di Monaco et al. (2016); Tayefi et al. (2018); Bo et al. (2020); Lee et al. (2020). This trend in using R for BP data sets is consistent with general popularity of R in data analyses Masuadi et al. (2021); Muenchen (2012), with Muenchen (2011) providing detailed guidelines and comparison of functionality for SAS and SPSS software users who wish to use R. Third, being free and open-source, R has an incredibly rich set of data analysis and graphics tools, with over 5,000 add-on packages available in addition to its base functionality. We are able to take advantage of this functionality in designing customized functions and visualizations. Concretely, software solutions like SAS and SPSS do not have customized functions for BP data, so all analyses are done using user-customized scripts. For example, a user interested in calculating dipping percentage will need to subset the data into sleep and wake periods, calculate mean values for each period, and then use the ratio of those values to calculate the Dip %. Since there is no built-in functionality to perform these steps simultaneously, they must be run sequentially, written from scratch by the user. In contrast, our R package bp combines general purpose R functionalities into functions that are of particular interest when analyzing blood pressure data. Using the above example, there is an explicit dip_calc function that performs all of the necessary steps, requiring only one line of code (more complex code is absorbed inside the function).

   We agree that it would be of great interest to compare custom bp functionalities like dipping calculation in dip_calc against other software solutions, however such cross-comparison is challenging due to the lack of custom BP functionalities in SAS and SPSS software, and the cost associated with obtaining proprietary BP software (e.g. AccuWin Pro^{TM} by SunTech Medical). Nevertheless, we hope that public availability of our example datasets together with underlying source code is a useful preliminary step toward this endeavor.

   In response to your comment, we have expanded our Introduction to highlight the benefits of R. We have also expanded our Discussion to highlight the limitations of custom validations since the majority of bp functionalities are not available in other statistical software.

3. *A significant problem with this program is the classification of BP values which is based on office BP that are applied to home and ambulatory BP that have different thresholds. Since they are statisticians, I recommend they seek the support of an expert in out-of-office BP analysis. They should also refer to significant guidelines such as those from ESH: home*

BP `https://doi.org/10.1097/HJH.0000000000002922` *ambulatory BP* `https://doi.org/10.1097/HJH.0000000000000221`

Thank you for bringing this very important issue. We acknowledge this is a significant problem with the previous version, and was not properly addressed. We have studied the ESH guidelines you pointed out in addition to ACC/AHA guidelines (Muntner et al., 2019). We acknowledge that our default threshold values for BP classification are based on thresholds designed for office BP, and we clarify this in the manuscript. We agree that in general these thresholds should be adjusted for out-of-office BP, however we discovered some variability in recommendations on how these adjustments should be made (as summarized in Tables 3-4 in Muntner et al. (2019) for ABPM and HBPM, respectively). For example, office BP thresholds of 120/80 correspond to HBPM thresholds of 120/80 according to ACC/AHA but thresholds of 120/75 according to IDHOCO outcome-based study (Niiranen et al., 2012). We found similar variability in correspondence of office BP thresholds to ABPM thresholds. When looking at extreme Low ($<$100 SBP or $<$60 DBP) and Crisis ($\geq$180 SBP or $\geq$120 DBP) categories, we were unable to find recommended threshold correspondence. Due to this variability, and that the focus of our work is on software rather than threshold recommendations, we have chosen to leave office BP threshold values as default implementation. However, we made significant modifications to the software to allow for both automatic thresholds adjustments based on BP type as well as full user customization of threshold choices. First, using 2017 ACC/AHA guidelines as references (we picked those over ESH due to some existing functionality relying on AHA recommendations), the software now automatically modifies thresholds for Stage 1 and Stage 2 Hypertension depending on whether HBPM or ABPM type is supplied whenever user selects `guidelines = "AHA"`. Second, we have included threshold values as optional parameters in processing and staging functions that can be fully customized by the user. These changes are reflected in detail in the updated software documentation, and are summarized in the main manuscript.

4. *I recommend moving the explanation regarding wSD to the section "Characterizing blood pressure variability," to which it better belongs.*

   Thank you for this suggestion; we agree that the background of the weighted Standard Deviation (wSD) is better suited in the Characterizing Blood Pressure Variability section and have moved the necessary content accordingly. We do believe that there is value is demonstrating its effects compared with traditional standard deviation and have decided to re-orient the code-specific content pertaining to wSD in the case study. We believe that by splitting this content up, as you suggested, the reader is better able to follow the case study without getting lost in the details of the metric itself.

R.1 Reviewer 2 comments

The work presented by Schwenck et al. addresses the unmet need for an open-source environment that is able to perform a large variety of blood pressure-related calculations in R independent from the device manufacturer. The manuscript is very well written, contains an adequate amount of references for all employed protocols and calculations and gives plenty of examples on how to use the software. I only have a few minor comments for the authors.

Thank you for your positive feedback.

1. *I might have missed it, but the open source license of the software should be mentioned in the manuscript.*

Thank you for pointing this out. Your suggestion has been incorporated into the manuscript in the introduction to mention our GPL-3 open source license.

2. *Code listings from e.g. from line 508 on could include code comment blocks such as "# here we load the data" or similar as this makes the blocks easier to read and may open the software to novice R users.*

   This is a great suggestion. We agree that this will help the reader to connect how the functions are described in the paper with how they are executed in R. We have made such adjustments to the manuscript and added comments in the verbatim environment within LaTeX to provide further clarity.

3. *For the final version of the manuscript it may also be beneficial to add some kind of label to code blocks in order to refer to them from within the text.*

   Thank you for your suggestion. We were able to find a solution so that each code block at the beginning now states **Code Block #**, and we refer to this number throughout the manuscript for clarity.

4. *Although this goes more in the direction of the software than the manuscript, but I wonder if the authors have plans to include import functions for data from specific devices, such as Omron (where a clumsy CSV export is available).*

   The included bp_jhs data mentioned in the first case study is actually from an Omron Evolv monitor obtained through the Omron mobile application. Importing directly into the R environment is definitely a possibility depending on the API configurations of the respective device / company. These configurations are left to the end user who is absolutely encouraged to extract his/her own data if they are able to make such integrations themselves. However, from a software development perspective, these implementations are currently out of the scope of this particular package as they generally require a commercial agreement to integrate at scale and / or may be prohibitively expensive. Future work could even consider a script/function that automatically inputs the requisite API credentials in order to ease the setup procedure, but currently there has been no action taken in this direction within the package itself so far. We acknowledge this in the updated discussion.

   In terms of data that is directly supplied in R (whether it be .csv, .Rdata, .xls, etc.), the bp package is intended to be used with practically any data format, assuming the 2 basic inputs (SBP and DBP) are specified during processing. The package will continually be updated should an unexpected format arise that gives error.

References

Ahmed, J., V. Ozorio, M. Farrant, and W. Van Der Merwe (2015). Ambulatory vs office blood pressure monitoring in renal transplant recipients. *The Journal of Clinical Hypertension 17*(1), 46–50.

Bo, Y., K.-O. Kwok, V. C.-H. Chung, C.-P. Yu, K. K.-F. Tsoi, S. Y.-S. Wong, and E. K.-P. Lee (2020). Short-term reproducibility of ambulatory blood pressure measurements: a systematic review and meta-analysis of 35 observational studies. *Journal of hypertension 38*(11), 2095.

Chang, W., J. Cheng, J. Allaire, Y. Xie, and J. McPherson (2020). *shiny: Web Application Framework for R.* R package version 1.4.0.2.

Di Monaco, S., F. Rabbia, M. Covella, C. Fulcheri, E. Berra, M. Pappaccogli, E. Perlo, C. Bertello, and F. Veglio (2016). Evaluation of a short home blood pressure measurement in an outpatient population of hypertensives. *Clinical and Experimental Hypertension 38*(8), 673–679.

Lee, H., Y. Yano, S. M. J. Cho, J. H. Park, S. Park, D. M. Lloyd-Jones, and H. C. Kim (2020). Cardiovascular risk of isolated systolic or diastolic hypertension in young adults. *Circulation 141*(22), 1778–1786.

Masuadi, E., M. Mohamud, M. Almutairi, A. Alsunaidi, A. K. Alswayed, and O. F. Aldhafeeri (2021). Trends in the usage of statistical software and their associated study designs in health sciences research: A bibliometric analysis. *Cureus 13*(1).

Muenchen, R. A. (2011). *R for SAS and SPSS users.* Springer.

Muenchen, R. A. (2012). The popularity of data analysis software. *URL http://r4stats.com/popularity*.

Muntner, P., R. M. Carey, K. Jamerson, J. T. Wright Jr, and P. K. Whelton (2019). Rationale for ambulatory and home blood pressure monitoring thresholds in the 2017 American College of Cardiology/American Heart Association Guideline. *Hypertension 73*(1), 33–38.

Niiranen, T. J., L. Thijs, K. Asayama, J. K. Johansson, T. Ohkubo, M. Kikuya, J. Boggia, A. Hozawa, E. Sandoya, G. S. Stergiou, et al. (2012). The international database of home blood pressure in relation to cardiovascular outcome (idhoco): moving from baseline characteristics to research perspectives. *Hypertension Research 35*(11), 1072–1079.

Tayefi, M., N. Shabani, M. Saberi-Karimian, M. Oladi, M. Mouhebati, Z. Farjami, Z. S. Hoseini, M. Mohammadi-Bajgyran, A. A. Mahmoudi, M. E. Doustabad, et al. (2018). Systolic and diastolic blood pressure percentiles by age and gender in northeastern iran. *Journal of the American Society of Hypertension 12*(12), e85–e91.
